# Effectiveness and Optical Quality of Topical 3.0% Diquafosol versus 0.05% Cyclosporine A in Dry Eye Patients following Cataract Surgery

**DOI:** 10.1155/2016/8150757

**Published:** 2016-02-16

**Authors:** Jang Hoon Lee, In Seok Song, Kyoung Lae Kim, Sam Young Yoon

**Affiliations:** ^1^Department of Ophthalmology, Kangdong Sacred Heart Hospital, Hallym University College of Medicine, Seoul 05355, Republic of Korea; ^2^Department of Ophthalmology, Hanyang University Medical Center, Seoul 04763, Republic of Korea

## Abstract

*Purpose.* To evaluate the effectiveness and optical quality of 3.0% topical diquafosol versus 0.05% cyclosporine A in dry eye patients following cataract surgery.* Methods.* In total, 40 eyes of 40 patients newly diagnosed with dry eye syndrome 1 week after cataract surgery were randomized to receive either 3.0% diquafosol ophthalmic solution six times daily or 0.05% cyclosporine A twice daily for 3 months. Outcome measures were tear film break-up time (TBUT), results on Schirmer 1 test, ocular surface staining score, the ocular surface disease index (OSDI) score, and higher-order aberrations (HOAs). Measurements were taken at baseline and at 1, 2, and 3 months.* Results.* In the diquafosol group, TBUT showed higher outcomes than the cyclosporine A group at 1 and 3 months. Both groups showed increased scores on Schirmer 1 test. The ocular surface staining score decreased in all periods in both groups. Vertical coma and total HOAs decreased more in the cyclosporine A group than in the diquafosol group at 3 months.* Conclusion.* Both 3.0% diquafosol and 0.05% cyclosporine A were effective in treating dry eye after cataract surgery. Diquafosol was more effective in increasing the tear secretion, but cyclosporine A was more effective in improving optical aberrations.

## 1. Introduction

Cataract surgery has undergone major advances in treating visual loss in cataract patients. For example, the smaller incision site of phacoemulsification decreases postoperative complications and shortens recovery time. It is currently assumed that the use of advanced technology in cataract surgery decreases postoperative symptoms. However, many patients still complain of irritation, blurring, and visual disturbances after surgery. A possible reason for this is dry eye caused by tear film instability after surgery [1, 2]. Dry eye not only affects the ocular surface, leading to irritable symptoms but also affects vision and overall quality of life. There are numerous causes of dry eye, including nerve cell injury during surgery, ocular epithelial injury due to corneal exposure, and use of conventional postoperative eye drops such as anti-inflammatory agents, topical corticosteroids, and anti-infectives [2–4].

Tear film instability during dry eye is the most common cause of decreased optical qualities of the eye. Previous studies suggested that this decrease is the primary cause of blurry vision associated with dry eye syndrome and tear film disruption [5]. Tear film changes in dry eyes may lead to irregularities of the corneal surface [6], and previous studies have reported that dry eyes can have an irregular tear film distribution across the cornea [7, 8].

To treat dry eye syndrome, numerous pharmacological eye drops have been used, including artificial tears, corticosteroids, autologous serum, sodium hyaluronate, and immunomodulators. As a commonly used eye drop, 0.05% cyclosporine A is a potent immunomodulatory agent that inhibits T-cell activation and downregulates the production of inflammatory cytokines, resulting in reduced surface inflammation [9–11]. Three percent diquafosol is a P2Y2 purinergic receptor agonist that activates P2Y2 receptors on the ocular surface [12]. Diquafosol stimulates both fluid secretion from the conjunctival epithelial cells and mucin secretion from the conjunctival goblet cells directly on the ocular surface, by interacting with the P2Y2 receptors to increase the tear film stability [12].

Previous studies have described the mechanisms and outcomes of these two topical eye drops [9, 11–14]. They differ in their mechanisms of action on dry eye, possibly leading to different outcomes. No previous study has compared and standardized the effects and optical quality of these two types of eye drops, but this would be valuable to optimize the treatment of dry eye patients after cataract surgery. Therefore, the objective of the current study was to compare the efficacy and safety of these two types of topical eye drops in stabilizing the tear film to treat dry eye patients following cataract surgery.

## 2. Material and Methods

### 2.1. Study Design

This study was a prospective, open-label, randomized, controlled study that followed the tenets of the Declaration of Helsinki and was approved by the Institutional Review Board at Kangdong Sacred Heart Hospital (agreement number 2015-04-002-006). After receiving an explanation of the nature and possible consequences of the study, all patients provided informed consent before being treated.

### 2.2. Patients

In total, 45 eyes of 45 patients newly diagnosed with dry eye syndrome 1 week after cataract surgery were enrolled in the study. They had undergone phacoemulsification and an intraocular lens implantation between April 2015 and June 2015. All surgeries were performed by a single surgeon. A monofocal aspheric, hydrophilic, acrylic intraocular lens with aberration neutrality (Akreos ADAPT AO, Baush & LOMB, USA) was implanted using an injector following ultrasonic emulsification. Subjects were aimed for emmetropia using an appropriate intraocular lens as measured by the SRK/T formula. None of the subjects were aimed for monovision in this study. After the operation, patients used topical antibiotics (Vigamox®, moxifloxacin hydrochloride; Alcon, Fort Worth, TX, USA) and a topical steroid (Pred Forte®, 1% prednisolone acetate; Allergan, Dublin, Ireland) four times a day for 5 weeks.

At 1 week after cataract surgery, only patients with mild to moderate dry eye syndrome were included in this study, involving an Oxford score of 1–3 and a TBUT of 3–9 seconds regarding the 2007 International Dry Eye Workshop criteria [15]. Patients with severe dry eye syndrome (Oxford score ≥ 4, and a TBUT ≤ 2 seconds), other diseases affecting tear film stability, or continuous dry eye medication previous to cataract surgery were excluded.

### 2.3. Randomization and Treatment Administration

Patients who provided informed consent were enrolled in the study by their treating physician and were assigned a sequential number with a corresponding randomization code generated by an independent third party using SAS software (version 8.0, SAS Institute Inc., Cary, NC). According to the randomization code, clinical staff assigned patients to receive either 3.0% diquafosol ophthalmic solution six times daily or 0.05% cyclosporine A twice daily from 1 week to 3 months after cataract surgery. The clinical staff provided treatment medications and instructions on how to administer ophthalmic solutions according to the assigned randomization group.

### 2.4. Outcome Measures

Patient's baseline characteristics were measured at 1 week after cataract surgery by an ophthalmological examination, with additional evaluation of ocular surface fluorescein staining (grades 0–5, according to the Oxford score), tear break-up time (TBUT), Schirmer 1 test results, and optical aberrations using a Hartmann-Shack wavefront aberrometer (WASCA; Carl Zeiss Meditec, Oberkochen, Germany). Optical aberrations were recorded in mesopic conditions without any pharmacologic mydriasis, were analyzed by expanding the set of Zernike polynomials, and were expressed for the central 4 mm diameter [16, 17]. Patients were reexamined at 1, 2, and 3 months after surgery, and the ocular surface disease index (OSDI) questionnaire was surveyed at each visit.

### 2.5. Statistical Analysis

SPSS for Windows software (ver. 18.0; SPSS, Chicago, IL, USA) was used for statistical analyses. Categorical data were analyzed using the Fisher's exact test. The changes in continuous variables from baseline were analyzed using the Wilcoxon signed rank test. The Mann-Whitney *U* test was used to compare changes in continuous variables between the two groups. A *P* value <0.05 was considered statistically significant.

## 3. Results

Forty eyes qualified and completed all study procedures. No intraoperative complications occurred in any of the surgeries (e.g., posterior capsular tear and vitreous loss). There was no patient presenting corneal edema at 1 week after surgery in both groups. The mean age was 64.3 ± 9.44 years in the diquafosol group (*n* = 20) and 63.4 ± 12.2 years in the cyclosporine A group (*n* = 20). Baseline clinical characteristics showed no differences in age, sex, spherical equivalent, OSDI, TBUT, Schirmer 1 test results, ocular surface staining scores, or ocular aberrations (Table 1).

**Figure 1:**
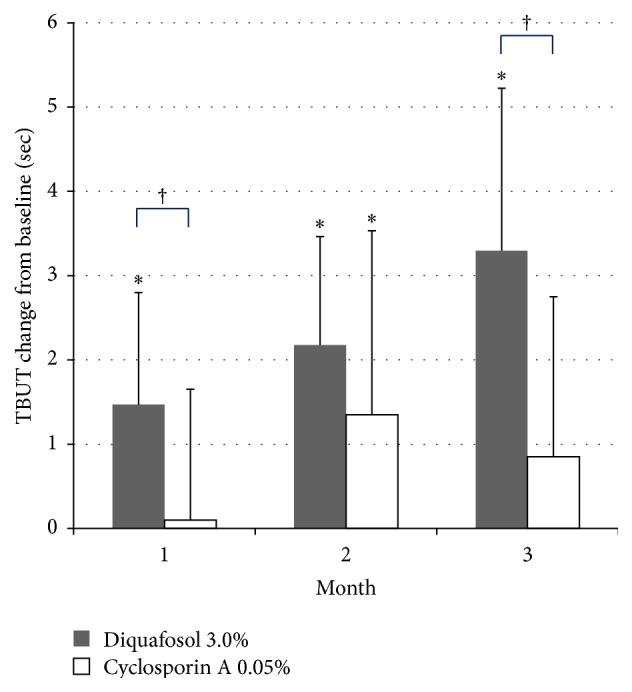
Change in tear break-up time (TBUT) from baseline. Mean value + standard deviation. *∗* = statistically significant difference in changes of TBUT from baseline (*P* < 0.05, Wilcoxon signed rank test). † = statistically significant difference in TBUT between the two groups (*P* < 0.05, Mann-Whitney *U* test).

**Figure 2:**
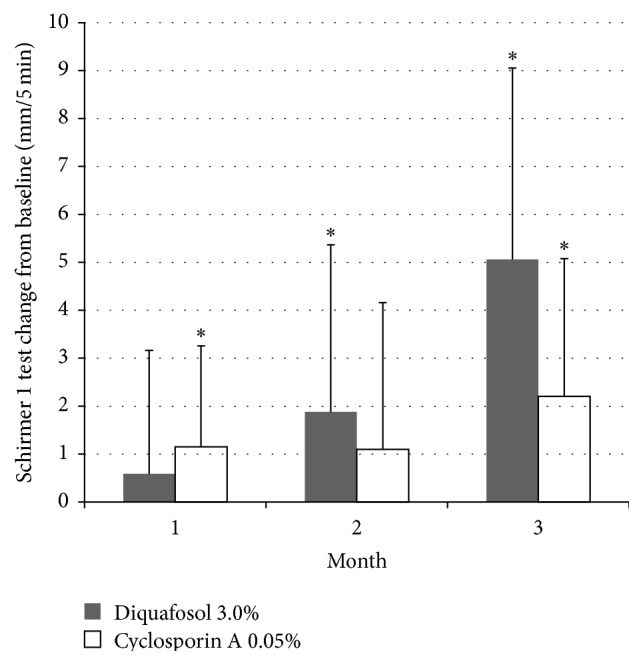
Change in Schirmer 1 test score from baseline. Mean value + standard deviation. *∗* = statistically significant difference in changes of Schirmer 1 test from baseline (*P* < 0.05, Wilcoxon signed rank test).

**Figure 3:**
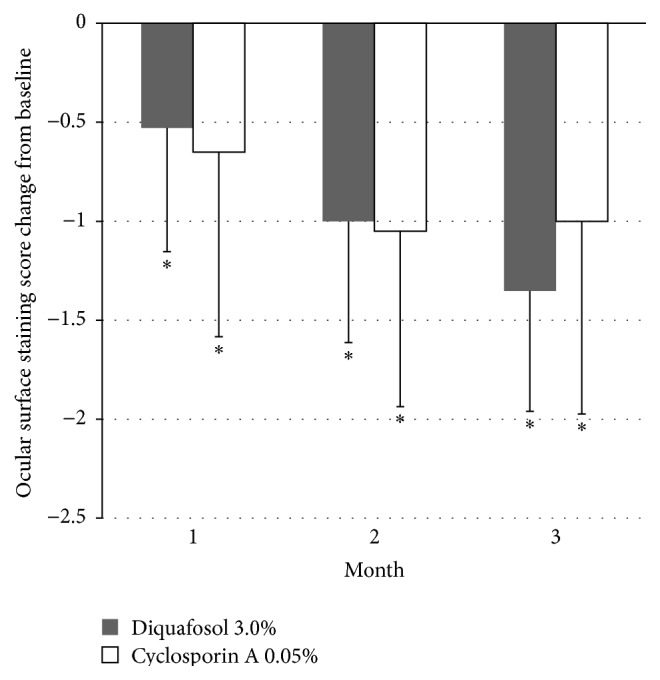
Change in ocular surface staining score from baseline. Mean value − standard deviation. *∗* = statistically significant difference in changes of ocular surface staining score from baseline (*P* < 0.05, Wilcoxon signed rank test).

**Figure 4:**
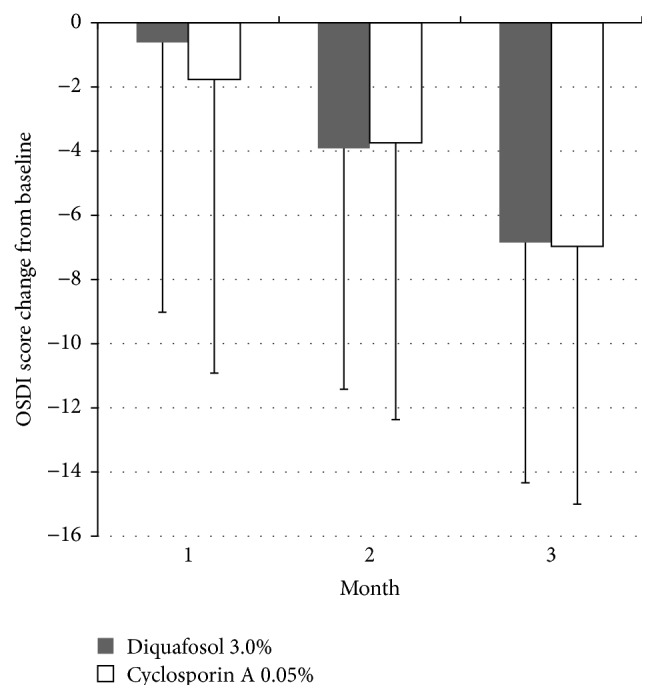
Change in the ocular surface disease index (OSDI) score from baseline. Mean value − standard deviation. All OSDI scores showed a decreasing pattern throughout the treatment period in both groups, but there is no statistically significant difference.

**Figure 5:**
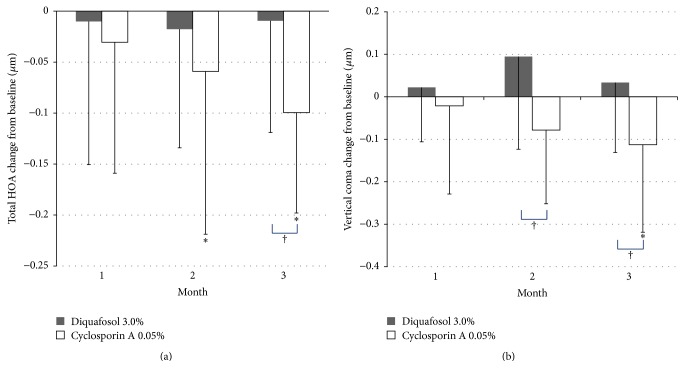
(a) Change in total higher-order aberrations (HOAs) from baseline. (b) Change in vertical coma from baseline. Mean value − standard deviation. *∗* = statistically significant difference in changes of aberrations from baseline (*P* < 0.05, Wilcoxon signed rank test). † = statistically significant difference in aberrations between the two groups (*P* < 0.05, Mann-Whitney *U* test).

TBUT showed improvement at 1, 2, and 3 months in the diquafosol group, but only at the second month in the cyclosporine A group (Figure 1). The diquafosol group showed better TBUT results at 1 month (*P* < 0.001) and 3 months (*P* = 0.001) when compared with the cyclosporine A group (Figure 1). Schirmer 1 test scores increased at 2 months (*P* < 0.05) and 3 months (*P* < 0.001) in the diquafosol group, and at 1 month (*P* < 0.05) and 3 months (*P* = 0.006) in the cyclosporine A group (Figure 2). The diquafosol group had higher scores on Schirmer 1 test at 3 months, although the group difference was not statistically significant (*P* = 0.06). Ocular surface staining showed improvement at all periods for both groups, but there was no significant difference between the two groups (Figure 3). All OSDI scores (e.g., symptom intensity, frequency, and aggravation) showed a decreasing pattern throughout the treatment period in both groups and failed to show a significant improvement (Figure 4). Total HOAs showed improvement in the cyclosporine A group at 2 months (*P* < 0.05) and 3 months (*P* = 0.002) and better results compared with the diquafosol group at 3 months (*P* < 0.05) (Figure 5(a)). Vertical coma showed an improvement in the cyclosporine A group at 3 months (*P* < 0.05) and a significant difference compared with the diquafosol group at 2 months (*P* < 0.01) and 3 months (*P* < 0.05) (Figure 5(b)). All other optical aberration values showed no significant changes.

## 4. Discussion

Dry eye syndrome is the most common disorder of the eye, and numerous theories of its pathogenesis, as well as numerous treatment options, have been reported. Dry eye after cataract surgery develops as a result of damage to the long ciliary nerves. Nerve injury leads to a decrease in blinking and tear production, resulting in permeability and metabolic abnormalities that result in dry eye [18, 19]. In addition, corneal incisions made during cataract surgery can release inflammatory mediators that reduce corneal sensitivity and tear film stability [15, 20]. Consistent with this theory, a previous study reported that OSDI, TBUT, and tear secretion decrease after cataract surgery [20]. Various treatments, especially cyclosporine A, have shown improvement of dry eye after cataract surgery. Cyclosporine A affects cytokine levels to control inflammation and has been shown to be an effective dry eye treatment, as assessed by Schirmer 1 test, the TBUT, and the OSDI [13]. The mechanism of action of cyclosporine A in dry eye is not completely known, but one possible mechanism involves the inhibition of cytokine production by activated T lymphocytes, resulting in reduced inflammation of the ocular surface and improved tear film stability [21, 22]. Consistent with this possibility, Jeon et al. reported that treatment with 0.05% cyclosporine A led to tear film stability. In this study, tear film instability was caused by inflammation due to corneal incisions, so controlling the inflammation led to improved stability [23]. Inflammation may therefore be a significant factor in tear film instability, leading to increased optical aberrations. There were some reports that improvement of tear film stability led to improvement in optical aberrations [17, 24]. In our study, 0.05% cyclosporine A treatment resulted in a significant decrease in optical aberrations due to a decrease of inflammation. Furthermore, 0.05% cyclosporine A treatment showed improvement at 2 and 3 months postoperatively, suggesting that improvement of optical aberrations by inflammatory modulators such as 0.05% cyclosporine A requires a relatively long period of continuous medication. In contrast, 3.0% diquafosol showed a rapid increase in TBUT and Schirmer 1 test results, which resulted in OSDI scores that reflected a decrease in dry eye symptoms.

The P2Y2 receptor agonist activates calcium secretion in the conjunctival epithelial and endoplasmic reticulum cells to increase secretion of mucin. Mucin performs an important role in protecting the surface of the cornea and stabilizing the tear film [25, 26]. In the current study, stabilized tear film led to increased TBUT and improvement in the Oxford scheme test grades. Furthermore, patients in our study showed rapid improvement of ocular surface dryness, leading to a decrease in dry eye symptoms. Although there was improvement in the TBUT and corneal staining, 3.0% diquafosol showed no significant decrease in optical aberrations. Meanwhile, there was no significant improvement in OSDI scores. Because the patients included in the study were limited to those with mild to moderate dry eye, it is possible that the improvement of symptoms did not significantly affect the scores.

Treatment with 3.0% diquafosol causes excessive secretion of fluid that paradoxically disturbs tear film stability. Choi and Shin reported that excessive secretion of tears (>10 mm) affected HOAs more than moderate secretion (6–10 mm) of tears [24]. The appropriate amounts of mucin and fluid stabilize the tear film, but excessive secretion due to continuous medication destabilizes this film. In addition, the P2Y2 receptor can play a role in the control of inflammation, so immediate treatment with 3.0% diquafosol after cataract surgery can affect inflammation [27]. In this study, the patients started using 3.0% diquafosol and 0.05% cyclosporine A 1 week after surgery, while using steroid eye drops for control of inflammation. Hyperemia, discharge, and anterior chamber reaction, which are regarded as symptoms of inflammation, were not observed in patients treated with 3.0% diquafosol. Also, there were no adverse effects in patients treated with 0.05% cyclosporine A.

There are some limitations to the present study. First, we enrolled patients with mild to moderate dry eye that met the 2007 International Dry Eye Workshop criteria. These means that our results should not be applied to all dry eye patients, in particular those requiring more intensive treatment. Also, this study was limited due to a small number of patients and the occasional lack of patient compliance. Five out of 45 patients failed to maintain the prescribed eye drop regimen and were excluded from the study (3 patients in the cyclosporine A group and 2 patients in the diquafosol group). Another factor interfering our study was that our aberrometric values were based on optical aberration. The optical aberrations are influenced by the tear film, the cornea, and the lenticular state. In particular, lenticular change such as posterior capsular opacification may affect the optical aberrations and interfere with the evaluation of the aberrometric values in dry eye. However, there was no patient with posterior capsular opacification throughout the follow-up period in this study. Although we did not observe any complications in all patients during follow-up period, the minimal positional change of IOL and capsule contracture may be correlated with HOAs values. Third, either 0.05% topical cyclosporine A or 3.0% diquafosol was additionally administered to the patients with the topical antibiotics and steroids during the same period from 1 to 5 weeks after surgery. In order to evaluate the effectiveness of the drug accurately, other drugs should not be used simultaneously or clearance period might be needed. However, we had focused on the additive treatment of the patient complaining of the symptoms of dry eye immediately after cataract surgery. Finally, this study is an open-label study using commercial eye drops. That means subjective factors cannot be excluded. The absence of blindness might affect the study results.

## 5. Conclusion

Both 3.0% diquafosol and 0.05% cyclosporine A were effective for the improvement of objective signs and subjective symptoms of dry eye after cataract surgery, but the timing and degree of therapeutic effects on tear film and ocular parameters differed between the two medications. Three percent diquafosol was more effective in increasing TBUT and Schirmer 1 test scores, while 0.05% cyclosporine A decreased optical aberrations.

## Figures and Tables

**Table 1:** Baseline characteristics in the diquafosol and cyclosporin group.

	Diquafosol (*n* = 20)	Cyclosporin A (*n* = 20)	*P*
Age (yrs)	64.3 ± 9.44	63.4 ± 12.2	0.892^*∗*^
Gender ratio (M/F)	0.67	0.67	0.750^†^
Spherical equivalent (D)	−0.75 ± 0.45	−0.5 ± 0.65	0.368^*∗*^
OSDI index (0–100)	20.1 ± 13.88	22.29 ± 13.66	0.798^*∗*^
TBUT (s)	3.17 ± 1.01	3.7 ± 1.08	0.141^*∗*^
Schirmer 1 test (mm/5 min)	7.7 ± 3.68	8.4 ± 3.60	0.278^*∗*^
Oxford score (0–5)	1.41 ± 0.62	1.5 ± 0.83	0.798^*∗*^
Total aberration (*µ*m)	1.00 ± 0.58	0.87 ± 0.35	0.684^*∗*^
Total HOAs (*µ*m)	0.33 ± 0.22	0.27 ± 0.10	0.752^*∗*^
Vertical coma (*µ*m)	0.20 ± 0.19	0.27 ± 0.22	0.357^*∗*^
Horizontal coma (*µ*m)	0.28 ± 0.21	0.32 ± 0.29	0.916^*∗*^
Vertical trefoil (*µ*m)	0.25 ± 0.19	0.23 ± 0.24	0.478^*∗*^
Oblique trefoil (*µ*m)	0.40 ± 0.34	0.29 ± 0.22	0.442^*∗*^
Spherical aberration (*µ*m)	0.20 ± 0.13	0.21 ± 0.14	0.707^*∗*^

D = diopter; M = male; F = female; OSDI = ocular surface disease index; TBUT = tear break-up time; HOAs = higher-order aberrations.

Mean ± standard deviation.

There was no statistically significant difference between 2 groups in baseline characteristics by ^*∗*^Mann-Whitney *U* test or ^†^Fisher's exact test (*P* > 0.05).
